# T-Cell Dependent Immunogenicity of Protein Therapeutics Pre-clinical Assessment and Mitigation–Updated Consensus and Review 2020

**DOI:** 10.3389/fimmu.2020.01301

**Published:** 2020-06-30

**Authors:** Vibha Jawa, Frances Terry, Jochem Gokemeijer, Shibani Mitra-Kaushik, Brian J. Roberts, Sophie Tourdot, Anne S. De Groot

**Affiliations:** ^1^Predictive and Clinical Immunogenicity, PPDM, Merck & Co., Kenilworth, NJ, United States; ^2^EpiVax, Inc., Providence, RI, United States; ^3^Discovery Biotherapeutics, Bristol-Myers Squibb, Cambridge, MA, United States; ^4^Biologics Development, Sanofi, Framingham, MA, United States; ^5^BioMedicine Design, Pfizer Inc., Andover, MA, United States; ^6^Center for Vaccines and Immunology, University of Georgia, Athens, GA, United States

**Keywords:** immunogenicity, protein therapeutic, T-cell, biologic, monoclonal, enzyme-replacement, anti-drug-antibody

## Abstract

Immune responses to protein and peptide drugs can alter or reduce their efficacy and may be associated with adverse effects. While anti-drug antibodies (ADA) are a standard clinical measure of protein therapeutic immunogenicity, T cell epitopes in the primary sequences of these drugs are the key drivers or modulators of ADA response, depending on the type of T cell response that is stimulated (e.g., T helper or Regulatory T cells, respectively). In a previous publication on T cell-dependent immunogenicity of biotherapeutics, we addressed mitigation efforts such as identifying and reducing the presence of T cell epitopes or T cell response to protein therapeutics prior to further development of the protein therapeutic for clinical use. Over the past 5 years, greater insight into the role of regulatory T cell epitopes and the conservation of T cell epitopes with self (beyond germline) has improved the preclinical assessment of immunogenic potential. In addition, impurities contained in therapeutic drug formulations such as host cell proteins have also attracted attention and become the focus of novel risk assessment methods. Target effects have come into focus, given the emergence of protein and peptide drugs that target immune receptors in immuno-oncology applications. Lastly, new modalities are entering the clinic, leading to the need to revise certain aspects of the preclinical immunogenicity assessment pathway. In addition to drugs that have multiple antibody-derived domains or non-antibody scaffolds, therapeutic drugs may now be introduced via viral vectors, cell-based constructs, or nucleic acid based therapeutics that may, in addition to delivering drug, also prime the immune system, driving immune response to the delivery vehicle as well as the encoded therapeutic, adding to the complexity of assessing immunogenicity risk. While it is challenging to keep pace with emerging methods for the preclinical assessment of protein therapeutics and new biologic therapeutic modalities, this collective compendium provides a guide to current best practices and new concepts in the field.

## Introduction

### Immunogenicity of Biotherapeutics: Historical Context

Immunogenicity is a term that is used in the biotherapeutic industry to describe undesired immune responses to protein or peptide drugs. Immunogenicity is driven by components that are intrinsic to the product (such as protein sequences integral to the drug itself), to host cell proteins that hitchhike along with the drug as it is purified, or to factors such as excipients that are related to drug formulation. Immunogenicity is also dependent on engagement of the individual patients' immune system and genetic factors that may pre-determine and shape their immune response.

**Figure 1:**
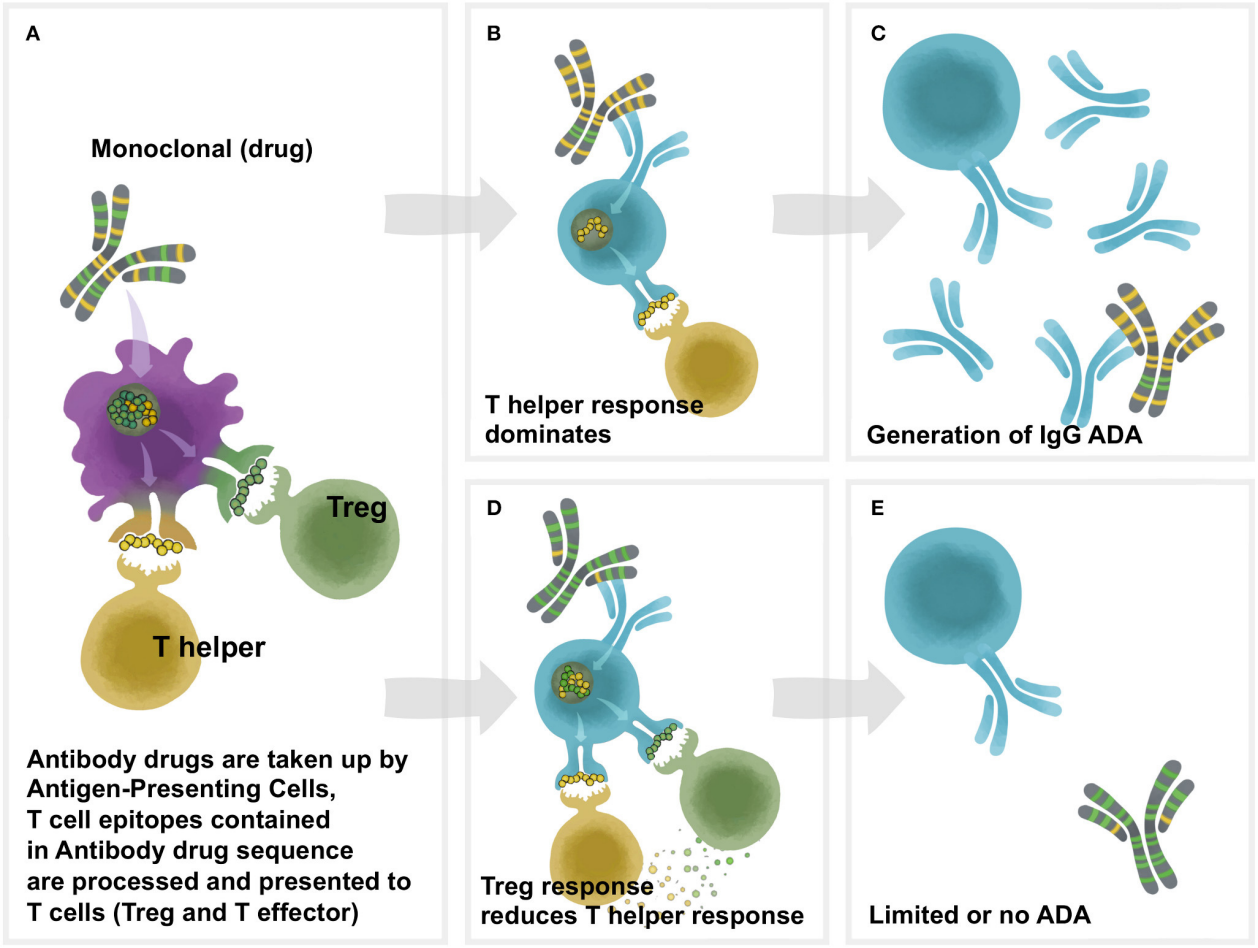
Overview of Td immunogenicity. T cell help is necessary for antidrug antibody formation. Proteins are phagocytosed by antigen-presenting cells (APC), after which they are degraded into peptide fragments and processed for presentation on class II HLA molecules on the surface of the APC. Peptides bound to HLA are recognized by cognate T cells which can be either effector or regulatory. Recognition of a regulatory peptide by a regulatory T cell promotes immune tolerance toward the protein, whereas recognition of an effector peptide by an effector T cell drives immunity. Upon antigen recognition, effector T cells are activated, leading to the activation of antigen-specific B cells which mature into antibody secreting plasma cells. In the absence of a T cell response, B cells are not activated and antibodies are not produced. **(A)** The framework and Fc region of monoclonal antibodies (and the sequences of other protein therapeutics) may contain different amounts of two types of T cell epitopes: Tregitopes, that activate natural regualatory T cells, and T helper (also known as T effector) epitopes, that are new to the human immune system and may engage helper, or effector T cells. These Helper and Treg cells help to modulate immune responses at the B cell level in the B cell follicle. In **(B)**, the lack of regulatory T cell epitopes leads to a T helper dominance and the development of antibodies (ADA) to the monoclonal as shown in **(C)**. As shown in **(D)**, T helper responses to the new epitopes in the CDR region are off-set by regulatory T cells that respond to Treg epitopes, leading to **(E)** lower levels of Td ADA.

While immunogenicity is often measured in terms of “anti-drug-antibodies” or ADA, obtained from clinical samples, the role of T cells that recognize drug-derived sequences presented on highly variable Human Leukocyte Antigens (HLA), is critically important to determining the immune response of any given subject (Figure 1). Both the individual patient's HLA haplotype and their personal B and T cell repertoire contribute to their individual immune response, leading to a high degree of patient-to-patient variability. Due to inherent variability in the immune systems of each individual patient (and, due to the imperfect means by which this response is measured), they may have no ADA at all, or they may have binding or neutralizing ADA that may reduce the efficacy of the drugs.

The immunogenicity to a therapeutic protein can be associated with hypersensitivity related reactions. Type 1 hypersensitivity is accompanied by ADA of the IgE isotype. Both IgE and high IgG ADA titers may contribute to significant adverse effects including infulsion reactions and/or anaphylaxis, although these types of adverse effects are uncommon. ADA-IgE complexes can bind and cross link the Fcϵ on basophils and mast cells, leading to IgE-mediated anaphylaxis. In addition, IgG ADA can complex with the therapeutic protein and these immune complexes can cross-link Fcγ receptors on neutrophils, releasing platelet activating factors that resemble histamine. Furthermore, large therapeutic-ADA complexes that fail to get cleared precipitate in the tissues like kidneys, synovial membrane and choroid plexus leading to tissue damage and organ failures (1, 2).

The most significant adverse events occur when ADA are cross-reactive with endogenous protein homologs. For instance, cases of pure red cell aplasia (PRCA) that were attributed to ADA developed unexpectedly after years of administration of recombinant erythropoietin (EPO) to patients without the development of any previous significant immunogenicity issue (3–6). These ADA were attributed to modification of the formulation and route of administration of EPO. PRCA was also recently observed during a clinical trial of a generic EPO developed by Novartis, and in this case was attributed to product aggregation induced by tungsten microparticles that were found in some lots of the drug product (4, 7). Other clinically significant adverse events related to ADA that cross-reacted with endogenous proteins include: neutralizing ADA caused by aggregates present in the formulation of human growth hormone (8), and ADA due to the presence of residual host cell proteins (HCP) in recombinant therapeutic products such as Factor VII (9).

These types of serious outcomes resulting from cross-reactive ADA have inspired the development of a wide range of *in vitro* methods for measuring the presence of ADA, which have been described in several white papers and regulatory guidance documents (10–17), including one on T-cell dependent immunogenicity published by our group in 2013 (19). In addition, methods for identifying drivers of immune responses to monoclonal antibodies and host cell proteins have also expanded and have been described in a number of publications (16, 20–29) and reviews (30) over the past few years.

As a result of these historical outcomes, regulatory agencies have asked drug developers to use a structured approach to measuring immunogenicity risk for biotherapeutics developers. For example, the European Medicines Agency (EMA) has published a “Guideline on Immunogenicity Assessment of Biotechnology-Derived Therapeutic Proteins” (17, 18) in which factors influencing the immunogenicity of therapeutic proteins were classified into helpful *patient-, disease-, or product-related* categories (see below). In addition to the EMA guidance, recent FDA guidelines for new drug products and generic versions of existing products have also suggested immunogenicity risk assessment approaches. See for example, the 2014 FDA guidance “Guidance for Industry: Immunogenicity Assessment for Therapeutic Protein Products”(31). This guidance highlights the contribution of T cell epitopes to immunogenicity and also mentions immune modulation attributed to regulatory T cells (22). Furthermore, many of the factors that might predispose a therapeutic protein to be immunogenic have been identified as “critical quality attributes” in the FDA-sponsored Quality-by-Design initiative (32) focused on manufacturing “process development.”

A recently published guidance for synthetic peptide drugs continues the regulatory guidance trend, expressly identifying the importance of T cell responses (33). Here, the Office of Generic Drugs at the FDA has suggested that immunogenicity assessment should extend to synthesis-related impurities, and asks peptide drug developers to evaluate whether impurities that may be co-purified with the active pharmaceutical ingredient (API) contain T-cell epitopes. These recommendations extend to five generic drugs but could be expanded to other novel peptide drugs, and to new generic drugs that enter the generic development pathway.

For peptide or protein-based drugs, the primary amino acid sequence itself can be a strong determinant of immunogenic potential. Beyond the primary sequence, agency guidelines point to *patient*- and *disease-related* categories that may pre-dispose a particular individual to an immune response (34). Examples include immune deficiency and concomitant immunosuppressive treatments such as methotrexate, which may decrease immunogenicity, and autoimmunity, which may increase the risk of ADA. In contrast, *product-related factors*, i.e., factors intrinsic to the final drug product itself that contribute to immunogenicity, may include modifications in the glycosylation profile (35–37), biophysical and biochemical attributes (10, 38–40), peptide manufacturing impurities and/or degradation products, or factors introduced during formulation (17, 28, 41, 42) Clearly, regulatory guidelines and updated preclinical immunogenicity risk assessment approaches are converging on a consensus, providing impetus for this review of the current state of the art.

### Focus on Td Immunogenicity Assessment and Mitigation

While immunogenicity is measured by testing for ADA, the root cause is T-cell dependent (Td) immune response, whether the driver is aggregates, host cell proteins, impurities, immune modulation due to target engagement or the sequence of the drug itself. Thus, **Td immunogenicity risk assessment** focuses on peptides known as T cell epitopes that may be derived from the sequence of the product (whether protein or peptide). Here we will focus on the biologic drug itself; host-cell proteins and other impurities that may be present in the drug product will be addressed in later sections.

Certain drug-derived peptides/epitopes may bind to human leukocyte antigen (HLA)/major histocompatibility complex (MHC) class II molecules, and the peptide/MHC complex is then presented to T cells on the APC cell surface (19, 22). More specifically, T cell epitopes that are processed and derived from the drug substance of the type known as *T helper* epitopes, are critically important to the development of ADA. The T helper epitopes are presented by a subset of HLA class II molecule (predominantly HLA DR but also DP or DQ) to CD4+ T cells which then provide the essential cytokines for B cell maturation and affinity maturation of the ADA. These interactions occur in the germinal center of lymphoid organs, where dendritic cells and B cells present T cell epitopes to T follicular helper cells and T follicular regulatory cells, which regulate the maturation of humoral immune response (43).

Just as identification of T helper epitopes is central to the process of immunogenicity risk assessment, removal of T cell epitopes; a process known as de-immunization, is key to Td immunogenicity risk mitigation. De-immunization is a process that is now entirely integrated into preclinical programs focused on mitigating Td immunogenicity risk. T cell epitopes that reduce immunogenicity, known as regulatory T cell epitopes, are equally important to immune responses to protein drugs that contain “human” components such as human-derived monoclonal antibodies, enzyme replacement therapies, and other human-origin biotherapeutics. Circulating regulatory T cells (Tregs) known as natural Tregs (nTregs) contribute to regulation of the human immune response and are also known to be epitope-specific (44, 45). The discovery of regulatory T cell epitopes known as Tregitopes in Immunoglobulin G (IgG) (46) improved risk assessment for monoclonals (47). The original IgG Tregitopes were published in Blood (22) others were published in Scientific Reports (48) IgG Tregitopes and “non-IgG” Tregitopes have also been identified in the patent literature (49). Discovery of regulatory T cell epitopes has now expanded beyond immunoglobulin, and is already improving the immunogenicity risk assessment of newer biotherapeutics as well.

A T-cell dependent immune response can drive an affinity matured anti-idiotypic response. Such a mature response driven by long term dosing can impact exposure, efficacy and safety as evidenced in enzyme replacement therapies and clotting factor proteins where immune response can not only lead to loss of exposure and efficacy but can have safety concerns due to cross reactivity to endogenous proteins or lack of other treatment alternatives. Some key examples of formation of neutralizing antibodies associated with loss of response are antibodies to FVIII/FVII and TNF inhibitors leading to loss of response (50–52). Safety concern key examples include development of IgE antibodies to cetuximab associated with anaphylaxis (53), antibodies to EPO associated with pure red cell aplasia (54) and antibodies to MGDF/TPO leading to thrombocytopenia (6).

In summary, a T cell-focused approach to the mitigation of immunogenicity emerged by 2010, leading experts to codify existing Td approaches to immunogenicity assessment and mitigation in the first version of this Td immunogenicity “white paper” (19). Almost a decade later, new concepts have emerged, and new modalities are in the clinic, and it is time to update and review Td immunogenicity.

### Definitions: T-Dependent Immune Responses to Biotherapeutics

#### Self vs. Non-self

Before addressing Td immune responses to biotherapeutics in further detail, it is helpful to remember that immune responses to these drugs can be divided into two broad categories. The first category would include what are considered to be “foreign” proteins (foreign to the patient), and the immune response to these proteins is typical of responses elicited against pathogens, vaccines, or allotypic antigens. Blood factors such as Factor VIII fall in this category since they are developed for individuals who are lacking, in whole or in part, the endogenous counterpart. This is also true for replacement enzymes such as acid alpha glucosidase (GAA), for Pompe disease. The second category of biotherapeutics involves autologous proteins (“self”), and thus “immunogenicity” to these proteins suggests a breach of B and/or T cell tolerance, similar to the response elicited to autologous self-proteins in certain autoimmune diseases.

**Figure 2:**
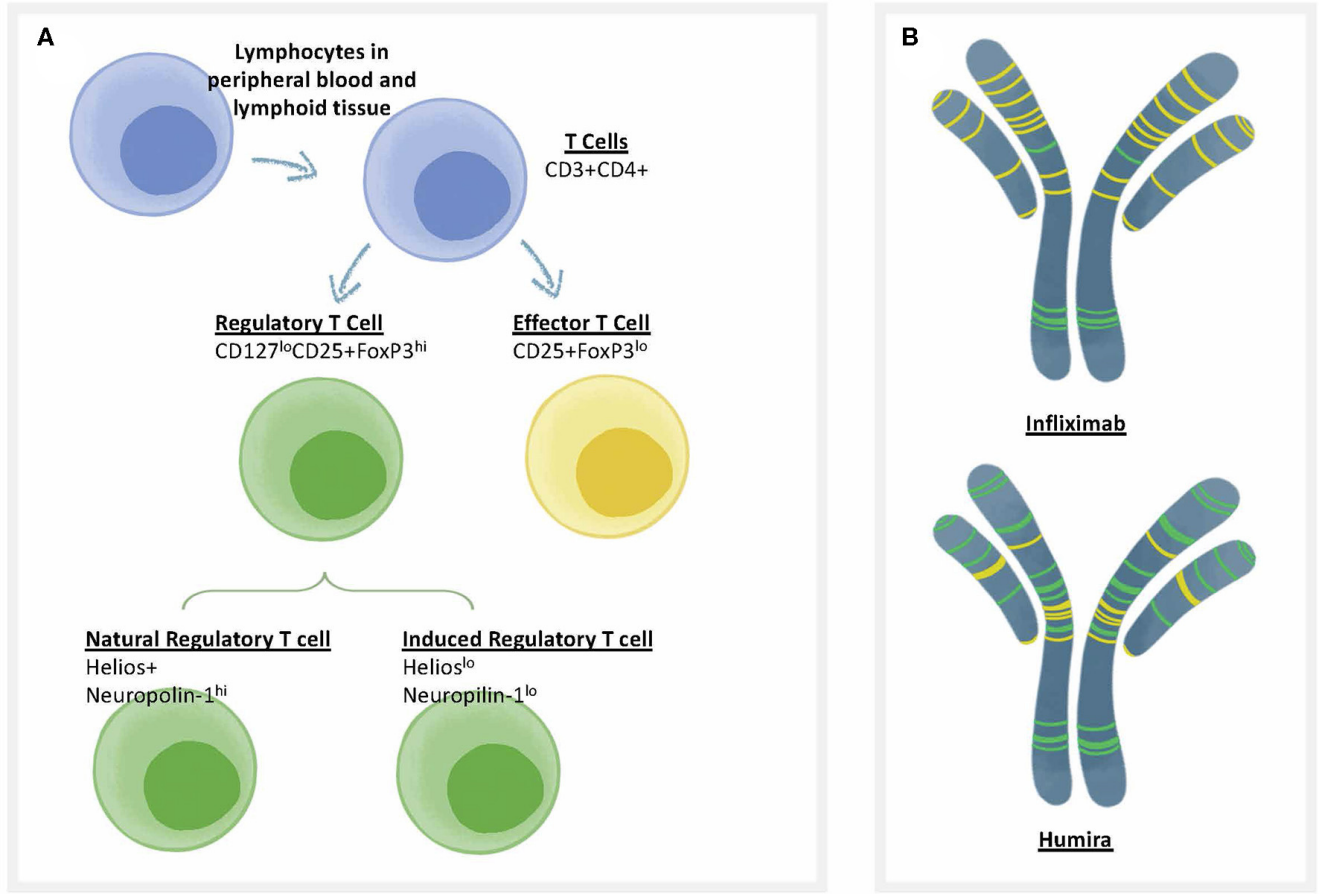
Treg epitopes, T effector epitopes, and T cell types. **(A)** Categories of T cells that are involved in immune responses to biotherapeutics. CD4 T cells (also known as T helper cells) orchestrate T-dependent anti-drug antibody responses. One of the best methods to differentiate the types of T cells that respond to biotherapeutics is to use flow cytometry, in which the surface and internal markers are used to differentiate categories that correlate with function. There are two types of T cells that can respond to biologics, including Regulatory T cells or “Tregs” (characterized by low levels of the cell surface marker CD127 and high levels of the internal marker FoxP3); and Effector T cells that are CD25 intermediate, FoxP3 low. Regulatory T cells can be further divided into natural regulatory T cells that are trained in the thymus, and induced (iTReg) Tregs that can be induced in the periphery. Each cell type has characteristic cell surface markers. **(B)** Categories of T cell epitopes found in biotherapeutics. The framework and Fc region of monoclonal antibodies may contain different amounts of two types of T cell epitopes: Tregitopes, that activate natural regualatory T cells, and T effector epitopes, that are new to the human immune system and may engage helper, or effector T cells. Two types of anti-TNF monoclonals are shown, with colored lines representing the approximate location of Treg epitopes found in their sequence in green and T effector epitopes in yellow [analysis by Rob Ventura of EpiVax, using the ISPRI Toolkit (157)].

Self-tolerance is actively regulated by circulating regulatory T cells (Figure 2A). These T cells respond to sequences in self proteins such as immunoglobulin, that may be identical in HLA binding features to non-self epitopes, but respond differently to activation of their T cell receptor (TCR). For example, regulatory T cells secreting IL-10 in response to HLA DR-restricted T cell epitopes in IgG have been identified by Franco and Sette (55) in immunoglobulin-treated subjects with Kawasaki's disease, and IL-10 responses (which may be due to Treg activation) have also been recorded in patients treated with infliximab to specific T cell epitopes derived from infliximab (56). Close inspection of peptide sequences eluted from antigen presenting cells that have been pulsed with monoclonal antibodies such as infliximab confirms the presence of many published and unpublished regulatory T cell epitopes known as Tregitopes, and these peptides do not elicit T cell responses (other than regulatory T cell responses) *in vitro* (57). See, for example, Figure 2B for an illustration of the location of Tregitopes (green) in two well-known monoclonal antibody drugs [infliximab (Remicade) and adalimumab (Humira)]; both monoclonals target TNF. Adalimumab (Humira) has fewer T cell epitopes and more Treg epitopes and is less immunogenic in the clinic (58).

Given that many self proteins such as monoclonals appear to contain regulatory T cell epitopes, the means by which breach of immune tolerance occurs is not as well-defined as the mechanisms for immune response to foreign proteins, but may include epitope mimicry, cross-reactivity of T cells, presence of trace levels of innate immune activators such as toll-like receptor agonists (42, 59, 60), and/or aggregated proteins (61). Genetic variations in Toll Like Receptors (TLR); polymorphisms in co-stimulatory molecules, modifications to cytokine receptors, and more, are likely to be involved in “breach of tolerance.” Patients who have autoimmune diseases may have some of these genetic anomalies and can be considered higher risk for developing ADA (62–65).

#### T-Independent vs. T-Dependent

Beyond regulation by T cell responses, humoral immune responses such as ADA can be thymus independent [T cell independent, (Ti)] rather than Td in origin (66, 67). For example, B cells may be activated in a Ti manner when particular structural patterns, such as polymeric repeats or carbohydrate molecules, directly activate B cells via the B Cell Receptor (BCR). Ti activation of B cells can be distinguished from Td activation, as the antibodies resulting from Ti activation are limited in both isotype and affinity and if memory B cells are generated, they are not long-lived (68, 69). In contrast, Td activation of B cells is characterized by class switching (IgM to IgG) and development of memory B cells that produce higher-affinity, more robust, and longer-lived antibody responses. The development of IgG-class antibodies following administration of a biotherapeutic generally indicates that the therapeutic is driving a Td immune response.

Td responses, by definition, are contingent upon T cell recognition of therapeutic protein-derived epitopes through the basic processes of protein antigen processing and presentation. Since human populations express a number of different HLA class II alleles, the interaction between antigenic epitope and HLA may exhibit a range of binding stabilities across the spectrum of HLA alleles expressed in the human population. This HLA genetic polymorphism and its consequent impact on the binding of specific peptides (HLA restriction) is the primary mechanism by which patient genetics (HLA haplotype) becomes a major determinant of immune responses to particular protein therapeutics.

#### Innate Immune Response

The innate immune system controls the initiation of Td immunity. Innate immune cells termed antigen presenting cells (APCs), upon activation in the periphery, migrate to the local lymph node where they can present drug-derived peptide antigens to antigen specific helper T cells in the presence of the proper co-stimulatory signals. Unlike the specific nature of the T and B cell receptors, cells of the innate immune system express germline encoded receptors termed pattern recognition receptors (PRRs) that recognize common microbial motifs (pathogen associated molecular patterns, or PAMPS). PRRs include several families of receptors such as Toll-like receptors (TLRs), RIG-1 helicases and C-type lectin receptors (42, 70).

In addition to recognizing microbial patterns, PRRs can also recognize a class of alarm signals called alarmins or danger associated molecular patterns (DAMPs) that are released in large quantities by stressed and dying cells to promote a localized inflammatory response. While DAMPs evolved to help combat pathogens, tissue damage, and stress that occurs during administration by a protein therapeutic can lead to DAMP-mediated inflammation and promotion of the adaptive immune response. Additionally, host cell and process derived impurities termed innate immune response modifying impurities (IIRMIs) can stimulate the innate immune system through interactions with PRRs promoting adaptive immunity. *In vitro* and *in vivo* studies have shown that IIRMIs, even at trace levels, can break tolerance to therapeutic proteins and promote an unwanted immune response (42).

New modalities such as Deoxyribonucleic acid (DNA) and ribonucleic acid (RNA) therapies, characterize the intersection between innate and Td immunogenicity in drug development. Firstly, these new delivery systems, may engage natural Toll-like receptors that are activated by RNA or DNA, and, second, they may be delivering a payload that is inherently immunogenic, not only by driving T help and antibody generation but also by driving adaptive T cell responses that eliminate the transduced target cells.

## New Concepts: Patient- and Drug-Specific Immunogenicity

Careful observation of the field over the past 5 years has contributed to the emergence of important new concepts in Td immunogenicity. These include observations related to the immune state of the patient receiving the biotherapeutic and the mechanism of action, or target of the biotherapeutic. For example, treatments targeting cardiovascular disease subjects are generally less likely to be associated with increased ADA, whereas autoimmune disease subjects may present with a spectrum of immune dysfunctions that can lead to increased propensity for anti-therapeutic response. Alternatively, some populations of patients may have unusual HLA distributions that are linked to greater presentation of T effector epitopes derived from the drug sequence, leading to higher or lower levels of immunogenicity. Lastly, the mechanism of action of the drug itself may interfere with, or promote, the activation of the immune system, leading to higher or lower risk of immunogenicity. Each of these topics is discussed in the next few sections.

### Patient-Specific Determinants of Immunogenicity

#### Disease Status

It is not uncommon to see one to two individuals per 100 that have higher baseline immune responses than others; these higher risk individuals may also have exaggerated immune responses to the delivery vehicle as well. The baseline immune status of a subject (including as described above, B and T cell repertoire as well as HLA hapolotype) can influence their ability to mount an immune response to a biologic. Tsang et al. (71) have established that such differences can influence the outcome of immune responses to the therapeutic proteins through an in-depth analysis of immune parameters associated with PBMC, frequency of cell populations, serum levels of chemokines and proteins indicative of immune activation. Also as described above, biotherapeutics may be more immunogenic in autoimmune disease patients due to the underlying inflammatory status of the recipient patient's immune system.

In years past, drugs that targeted patients who have auto-immune diseases included anti-TNF agents, which had remarkably different immune profiles in selected patient populations. A systematic review by Thomas (72) illustrates the variability of biotherapeutics in the context of Rheumatoid Arthritis (RA): the most immunogenic were infliximab (25.3%), followed by adalimumab (14.1%) and certolizumab (6.9%). These rates of immunogenicity are significantly higher than those reported for the same drugs in patients who have ankylosing spondylitis, which may either reflect the immune status of the patients or the HLA-skewing of select auto-immune diseases.

Explanations for the increased level of ADA in RA and autoimmune patients vary, however, such patients may have defective regulatory T cells (73–76) or lack functional regulatory T cell cytokine receptors (IL-2, IL-10) (77–79). Perturbation in the function of regulatory T cells or of regulatory cytokines that are critical for Treg function, may dramatically decrease Treg response to drugs that contain Tregitopes, which include many of the monoclonals that are used to treat autoimmune diseases. Drugs such as methotrexate and TNF-inhibitors have been shown to restore Treg function, potentially reducing ADA once the drug is at therapeutic levels (80). This is one potential explanation for the observation that ADA tend to be higher in patients who have, active, flaring RA; and may also explain why ADA may disappear with effective anti-inflammatory drug treatment (81).

Clearly, the immune system can be modulated by anti-inflammatory treatments (see also Tolerance induction section). Clinicians and drug developers may benefit from collaboration so as to improve the proactive assessment of immunogenicity in the context of autoimmune disease. Collaboration will enable personalized treatments and better clinical decisions based on improved awareness and detection of immunogenicity risk factors.

#### HLA

ADA measurement has further limitations with lack of reliability during dosing timepoints. The most optimal way to support the translatability of the algorithm and T cell based predictive assays is to correlate with T cell responses in the dosed donors. Several recent studies have shown the direct association of a mature ADA response with presence of therapeutic specific T helper cells (82–84). The algorithms used to identify sequence-based risks can provide the first glimpse of HLA types in a population that would be at risk to bind the non-self epitopes in a protein. Based on the prevalence of the HLA types for a geographical location, the risk for immunogenicity in clinic can be modeled. Additionally, HLA typing of subjects being enrolled for clinical trials can help track if the ADA responses are associated with the HLA that were predicted to be at risk. Indeed, the past decade has been marked by a flurry of publications related to the association of certain HLA class II alleles with immunogenicity risk for selected biologic therapeutics. Buck et al. (85) demonstrated that HLA-DRB1^*^04:01 and HLA-DRB1^*^07:01 multiple sclerosis patients exhibit an increased risk for developing neutralizing antibodies to IFNβ. A similar association of HLA-DRB1^*^04:01 and HLA DRB1^*^15 carriage with a higher risk of ADA development to IFNβ-1b and IFNβ-1a, respectively, was identified by Link et al. (86). Increased risk of ADA development to infliximab was also observed for HLA-DRB-11, HLA-DQ-03, and HLA-DQ-05 carriers in rheumatoid diseases (87) and HLA-DRB1^*^03 inflammatory bowel disease patients (88) and two risk alleles (HLA-DRB1^*^03 and HLA-DRB1^*^011) and three protective alleles (HLA-DQB1^*^05, HLA-DRB1^*^01, and HLA-DRB1^*^07) were described for various anti-TNFa in rheumatoid diseases patients (88, 89). Larger databases of patient data may reveal greater numbers of HLA-associations and may also simply confirm that all HLA class II molecules, rather than just one or two, perform the critical function of presenting T cell epitopes to the immune system in drug-exposed subjects.

#### Microbiome

Recent studies have confirmed the long-standing hypothesis that the human gut is inhabited by microbiota that can have a strong impact on host immune responsiveness. On the one hand, the immune system, including T cells that may bear TCR for novel epitopes found in biotherapeutics may be tolerized to the commensal pathogens due to presence of the Toll-like receptors (TLRs) on the epithelial and lymphoid cells of the small intestine that suppress any inflammatory responses and maintain intestinal homeostasis (90). On the other hand, the microbiota in the gut can influence the differentiation of the Th cell subsets that maintain homeostasis. In addition, NOD like receptors (NLRs) can also recognize the microbial organisms and modulate the immune responses of T cells to avoid inflammation. If the therapeutic T cell epitope sequence contains sequences that resemble sequences from the genome of the microbiota, the risk of mounting an immune response may be higher (if T effector epitopes are conserved) or lower if regulatory T cell epitopes are conserved with the drug (91). The influence of gut microbiota in individuals from geographical regions with a higher exposure to environmental pathogens vs. those from urban environments, and in individuals who have taken antibiotics prior to being treated with biologic therapeutics certainly deserves careful consideration by the immunogenicity risk assessment community.

#### Drug Function as a Determinant of Immunogenicity

With the emergence of immune-system-targeting biotherapeutics, it has become clear that the actions of the drug itself can also contribute to, or modulate immunogenicity. This was posulated to play a role in the activity of anti-TNF agents due to the impact of TNF on regulatory T cells, as described above. Improved Treg function as a result of anti-TNF therapy may lead to reductions in ADA to anti-TNF agents over the course of time (81, 92). Similarly, IL-2, a cytokine that is required for the function of regulatory T cells may not only induce a pro-regulatory environment but could also reduce the likelihood of ADA developing to the drug. This mechanism may contribute to the effectiveness of low-dose IL-2 therapy in autoimmune disease (93). Conversely, IL-2 is also capable of enhancing the function of effector T cell responses and has been used at high doses in the treatment of viral and oncological disease (94).

Teraparatide, a peptide drug, provides yet another illustration of target effects. It elicits cytokine release from T cells (e.g., TNFα, IL-1, and IL-6) as well as IL-2 (95, 96). Thus, Teriparatide may exert a direct effect (both pro-inflammatory and anti-inflammatory) on the immune system. And check point inhibitors, the newest class of biotherapeutics to hit the clinic, can directly interfere with immune response and contribute to immune response, potentially increasing immunogenicity as described in the next two sections.

#### Drug Target and Immunogenicity: Checkpoint Inhibitors

Some drugs, such as check point inhibitors (CPI), are used to enhance immune responses, As a result, checkpoint inhibitors have been proven to be successful in the treatment of aggressive cancers, and some of them are also more immunogenic than expected, potentially leading to loss of efficacy with continued treatments. One hypothesis is that their actions reduce the tolerizing effect of natural Tregitopes that may be present in the sequence of the checkpoint inhibitor drug and/or enhance effector T cell responses to foreign epitopes in the drug sequence. In line with the inhibition of immune inhibitory pathways, Treg depletion and a toxicology profile of decreased self-tolerance that is observed with CPI treatment, selected checkpoint inhibitors Atezolizumab (anti PD-L1) are associated with markedly higher ADA (39.1–48%) than would normally be expected given their fully human IgG framework.

The enhancing effect on immunogenicity appears to be especially salient when the drugs are used in combination. For example, the immune response to Nivolumab in monotherapy was 12%, however it was significantly increased to 24–38% when Nivolumab was dosed in combination with Ipilimumab (97). Combination therapies with checkpoint inhibitors like Pembrolizumab (Keytruda) and Nivolumab (Opdivo) and small and large molecule T cell modulatory targets like CTLA 4, Lag3, TIGIT, GITR, etc. have not only shown improved efficacy as noticed by tumor regression and long term survival but may also have the potential for demonstrated synergistic immunogenicity when used in combination (98–103).

Despite these observations, some checkpoint inhibitor monotherapies have demonstrated standard rates of immunogenicity (1–10% ADA); for example (97). The reason for these differences is as yet unexplained, but may be due to differing degrees of “intolerance” specific to the actions of the molecule that is the target of the CPI, as well as attributes that improve processing and presentation of the drug itself, in the inflamed tumor or draining lymph nodes. Technical limitations of ADA assays (104–106) might also contribute to differences in ADA incidences in the immuno-oncology field. In summary, while checkpoint therapeutics may reduce tolerance to tumors, they also appear to enhance the likelihood of T-cell driven immune response of the biotherapeutics especially when administered in combination.

#### Drug Target and Immunogenicity: Anti-inflammatory Cytokine Inhibition

In contrast with CPI, certain anti-cytokine agents are known to be much less immunogenic than expected. One such drug is an anti-IL-6 biologic, known as Tocilizumab, a drug that is now widely used in RA and in other autoimmune diseases (107). Notably, IL-6 is required for T cell activation, thus, interference with IL-6 may reduce T engagement and thereby reduce ADA. Another example of a drug that may directly interfere with immunogenicity is Rituximab, which targets CD20 on developing B cells and reduces the formation of antibody secreting plasma cells, which may explain why ADA are not generally detected for this drug.

## New Modalities

New means of delivering drugs such as via gene therapy (DNA, RNA) or encoded in a vector for delivery (108) may engage new types of immune response. For example, unexpected anti-drug CD8 T cell (HLA Class I-restricted) responses to biotherapeutics have been described recently. Specifically, therapeutic anti-CD19 CAR-T cells were destroyed by CD8 T cells that targeted murine sequence-derived T cell epitopes in the transgene, abrogating the efficacy of the CAR-T for several patients (109). Drugs that enter cells and are expressed by them (such as viral-vector mediated monoclonal antibodies) may be interfacing with cell mediated immune responses leading to unanticipated immunogenicity and, potentially, failure in the clinic.

### Biologic Therapy by Viral Vector

Next generation viral and cell-based therapies are now being diverted from the gene therapy market to deliver modalities that target solid tumors directly. Additionally, antibody drug conjugates (ADC) which are antibodies or alterative scaffolds, delivering small molecules like toxins or cell inhibitors conjugated to antibodies, are being used to target tumors. In addition, viral mediated transduction of antibodies and cytokines is being used to express the foreign transgenes in relevant cells, to enhance T-cell mediated killing. When viral vectors are used to deliver drugs, the impact is similar to a viral infection, engaging both CD8 T cell responses as well as CD4 T cell and antibody responses. Furthermore, the products may not be entirely pure, and thus hostcell proteins or impurities may be responsible for driving the immune response, not the sequence of the drug itself.

### Gene Therapy

Immunogenicity to gene therapy can be challenging to address, and has the potential to limit efficacy. Viral-based delivery may be intrinsically immunogenic because they contain T cell epitopes that drive T-cell mediated elimination of transduced cells, as was the case with adeno-associated vectors (AAV) (110). Both pre-existing antibodies or T cell responses to the viral delivery vector can neutralize the delivery of the viral vectors, and some clinical studies have exclusion criteria based on pre-existing anti-vector antibodies. Switching to different viral isotypes or engineering of the viral vector surface proteins is further complicated by the tissue selectivity of the vector, which may also be required for effective gene delivery (111).

The transgene [the intended drug product, such as a monoclonal antibody or a replacement protein (blood factor, other) which will be expressed in the patient's body] can also be the target of immunogenicity, and immunogenicity is not limited to ADA, especially if the transgene is intended to replace a defective (or absent) gene, which may lead to recognition of the transgene as a foreign protein. T cell responses to the transgene can include HLA class I mediated CTL response to the intracellular product of the gene therapy, damaging the tissue that expresses the transgene and leading to loss of functional gene therapy product (112). Thus, consideration of both HLA class I and HLA class II-restricted epitopes is required for immunogenicity assessment of the gene therapy vector and its transgene product.

### T Cell Specific Oncolytic Viruses

Oncolytic viruses are administered in combination with other therapeutic proteins like checkpoint inhibitors or immune modulatory targets to actively support tumor killing by activating the immune system (113). The efficacy of oncolytic viruses can however be impacted by development of neutralizing immune response to viral capsids as well as a virus specific T-cell response. Pre-existing immune response to the oncolytic virus can reduce the efficacy of the oncolytic virus due to neutralization by anti-viral antibodies, post-dosing (114, 115).

### Chimeric Antigen Receptor (CAR)-T

Autologous T cells that have been transduced with genes that express anti-tumor-specific antigens such as CD19 have been demonstrated to have significant antitumor activity in hematologic malignancies. Even though cell therapies have gained approval by US and European regulatory agencies, there are considerable immunogenicity challenges that arise during the production and administration of these personalized therapies. Both humoral and cell mediated responses can occur against unique chimeric antigen receptor (CAR) components (108). For example, immune response may target the CAR-T due to the presence of non-human sequences in the CAR construct and suicide domain components. Immunogenicity may also be generated by residual impurities such as viral proteins or other gene editing-related non-human proteins.

CD8+ T cell–mediated immune responses have been reported after anti-CD19 CAR–T cell infusion in some patients (116). These CD8 T cell response to CAR transgene limited CAR–T cell persistence and increased the risk of relapse. In the published study, five patients that had developed persistent leukemia or relapse after an initial infusion of anti-CD19 CAR-T received a second infusion of CAR-T cells, and for these patients, there was no expansion or persistence of CAR-T cells or demonstrable antitumor activity and infusion was followed by the loss of CAR T cell population. The loss was attributed to a specific CD8 T cell response to the CAR-T; a T cell line generated from one patient showed specific CD8+ restricted autologous CAR-T cell lysis which was shown to be driven by the murine portion of the CAR-T with a peptide ELISPOT (116).

### Peptide Drugs: Novel, Generic, and Peptide Impurities

Over the last several decades, important advances in peptide synthesis has contributed to a major shift in the manufacturing of therapeutic peptide drugs and an expansion in the number of novel peptides entering clinical pipelines. As for monoclonals, blood factors, and recombinant enzymes, HLA-binding sequences that are present in peptide drugs may activate regulatory or effector T cells, and therefore, peptides can be immunogenic in clinical use. The transition from fully recombinant to synthetic peptide drugs has led to increased regulatory concern about synthesis-related impurities that may induce unwanted immune responses including ADA. Regulatory experience with selected generic peptides has contributed to the development of draft guidelines for generic peptide products that was recently introduced by the Office of Generic Drugs at the FDA (33).

Immunogenicity to peptide drugs is primarily related to peptide synthesis methods that can introduce peptide impurities that may be difficult to remove from the final drug formulation. These impurities may contain novel T cell epitopes that could contribute to T cell activation (and ADA). In some cases, impurities have been associated with anaphylaxis (117). Several classes of peptide impurities can be generated at each step of the peptide synthesis process including amino acid insertions and deletions, incorporation of diastereomeric amino acids, and oxidation of amino acid R groups. In addition, impurities can arise during storage. A thorough review of impurities in peptide drugs, and where they occur in the synthesis process can be found in D'Hondt et al. “Related Impurities in Peptide Medicine (118). Analysis of these impurities can be performed with *in silico* tools and *in vitro* assays, similar to the process described below for biotherapeutics.

Relative to T cell dependent immunogenicity, new T cell epitopes may be introduced when unintended modifications to the amino acid sequence of the drug result in impurities that contain new HLA-binding ligands or changes to the TCR-facing contours of existing epitopes. For example, a novel GLP-1 inhibitor that was in commercial development was discontinued after the number of patients with confirmed positive anti-drug antibody tests increased from 16% at week 12 to 39% at week 24 (117); up to 5% of patients also developed systemic allergic reactions.

## Computational Immunogenicity Risk Assessment

### *In silico* Screening

Current practice of immunogenicity screening generally starts with an *in silico* assessment and then proceeds to HLA binding assays, T cell assays, and MHC associated peptide proteomics (MAPPs) as needed. Some groups (57, 83, 119) start with MAPPs and do not use *in silico* tools, however, MAPPS is resource-consuming and costly. Greater experience with and familiarity with available *in silico* tools is likely to lead to greater adaptation of these tools as the first step in immunogenicity assessment in the future. This section will briefly describe available tools and highlight improvements to these tools.

### T Cell Epitope Prediction

As described in section Definitions: T-Dependent Immune Responses to Biotherapeutics, ADA responses develop due to an adaptive immune response, supported by T cells responding to linear peptide epitopes displayed by HLA on the surface of APCs. For biotherapeutics delivered via conventional (exogenous, i.e., intravenous, subcutaneous, even topical) routes, presentation through the Class II pathway to CD4+ helper T cells is most relevant, however, as also discussed above, CD8+ T cell response biotherapeutics delivered by viral vectors and cell therapies is a rising concern. Fortunately, T cell epitopes can now be predicted with a high degree of confidence (A separate manuscript describing the typical approach to *in silico* risk assessment in detail has been submitted to this issue and topic in Frontiers).

The core residues of a T cell epitope sequence that define the affinity and stability of binding to pockets of HLA DR, DP and DQ alleles are generally nine amino acids long. Despite this fact, due to the open ended conformation of the Class II HLA binding groove, and the stabilizing effect of “flanking” residues around 9-mer core sequences, peptides reported to bind to Class II HLA and to stimulate T cell response are most often longer in length, and most web-accessible T cell epitope mapping tools parse full protein sequences into overlapping frames of 9–15 residues and report a rank, score or predicted affinity for each frame. Methods to assess the immunogenic potential of a complete protein are available on several public and academic platforms (120, 121) in some cases paired with mathematical models based on hypothetical binding affinities and T cell precursor frequencies, or with MAPPs-determined peptidomes (122–125).

Publicly available websites for epitope scanning may appear and disappear, and can also be modified, often without notification, leading to changes in immunogenicity interpretations over time. For this reason, many mid to large-pharmaceutical companies import on-line algorithms and operate them within their firewalls to reduce the risk of intellectual property disclosure. Others use web-based tools such as the secure-access commercial-grade ISPRI toolkit. Alternatively, companies may outsource immunogenicity prediction to commercial research organizations.

Some tools such as the commercial ISPRI platform use unique algorithms and knowledge to identify Treg epitopes in monoclonal antibody sequences and provide a statistical assessment of epitope content relative to random expectations and adjusted for selfness (i.e., tolerogenic potential) (126). Direct ranking of a new biologic drug products against other known non-immunogenic and immunogenic products is possible using a normalized “immunogenicity scale.” The toolkit also features novel algorithms to search for epitope that are “human-like” (see next section) and therefore less likely to engage activated T cells, and methods for deimmunization and tolerization that can be performed directly *in silico* (127).

### Screening for Self-Ness

T cells recognize not only peptide sequences, but the complex of peptide bound in the cleft of an HLA molecule. In any HLA ligand, certain amino acids are in contact with the HLA molecule itself, while others are accessible to the TCR. If TCR-facing residues from a given epitope are conserved among multiple HLA-binding sequences from the human proteome, the epitope in question may activate T cells specific to these human proteins. This may lead to a regulatory response generated by natural Tregs or to a limited or null response due to T cell anergy or deletion during thymic selection.

For many HLA alleles, the peptide positions responsible for anchoring in the HLA binding cleft are known, and other residues have been reported to interact with the TCR. Algorithms such as JanusMatrix (128) can be employed to screen predicted epitopes derived from candidate therapeutics against the human proteome to distinguish the peptides that are more self-like, and thereby likely to be tolerated, from those that have limited human cross-conservation and are thereby more likely to be recognized as foreign by the human immune system. Therapeutic-derived epitopes that appear foreign are the most likely targets of anti-therapeutic T cell response.

#### Screening Against Relevant Peptide Libraries

Once T cell epiotopes are identified, it is also possible to determine whether the epitope has been tested *in vitro* or *in vivo*. The Immune Epitope Database (www.iedb.org), a contracted endeavor from the US National Institute of Allergy and Infectious Diseases, has now curated 20,860 journal articles and direct submissions, cataloging nearly 622,105 peptidic epitopes (129). By screening novel sequences against this database, researchers can determine whether peptides related to the epitopes in products in development have been reported as MHC ligands, and whether the phenotype of T cell response is known, allowing for triage of well-understood sequences from unknown sequences of greater immunogenic risk. Furthermore, when risk signals are identified, proteomics databases that contain sequences elulted from antigen-presenting cells (130) can reveal important relationships across tissues and disease states to inform careful monitoring during clinical studies.

### Ranking Biologic Candidates by Immunogenic Potential

All other factors being equal, the greater the burden of T cell epitopes contained in a given protein, the more likely it is that the protein will induce an immune response. The comparison of one biologic to another is possible to accomplish by normalizing epitope content scores across HLA alleles and adjusting for sequence lengths, as is done on the ISPRI toolkit (47, 127). Regional epitope density can also drive immune responses. A detailed description of the global and regional approach to determining immunogenicity risk is described in detail in reference (47).

## *In Vitro* Methods for Assessing Immunogenicity Risk

Extensive validation *in vitro* assays may be cost-prohibitive, thus current practice is to initiate the analysis with advanced in silico tools (127). Following *in silico* analysis, HLA binding and T cell assays can be performed or outsourced to commercial research organizations. These assays can be applied (i) at the very early stages of drug development to design *de novo* therapeutics with low predicted immunogenicity, (ii) at a later stage to de-immunize a clinical asset exhibiting high immunogenicity in First *in Human* studies, (iii) retrospectively after program termination, to decipher the mechanisms and immunogenicity risk factors underlying the high observed clinical immunogenicity. Clearly, for new (and generic versions of older) biologic drugs to be successful, immunogenicity risk assessment is most cost-effective if performed in the pre-clinical phase of development.

### *In vitro* Assays

#### HLA Binding Assay

**Figure 3:**
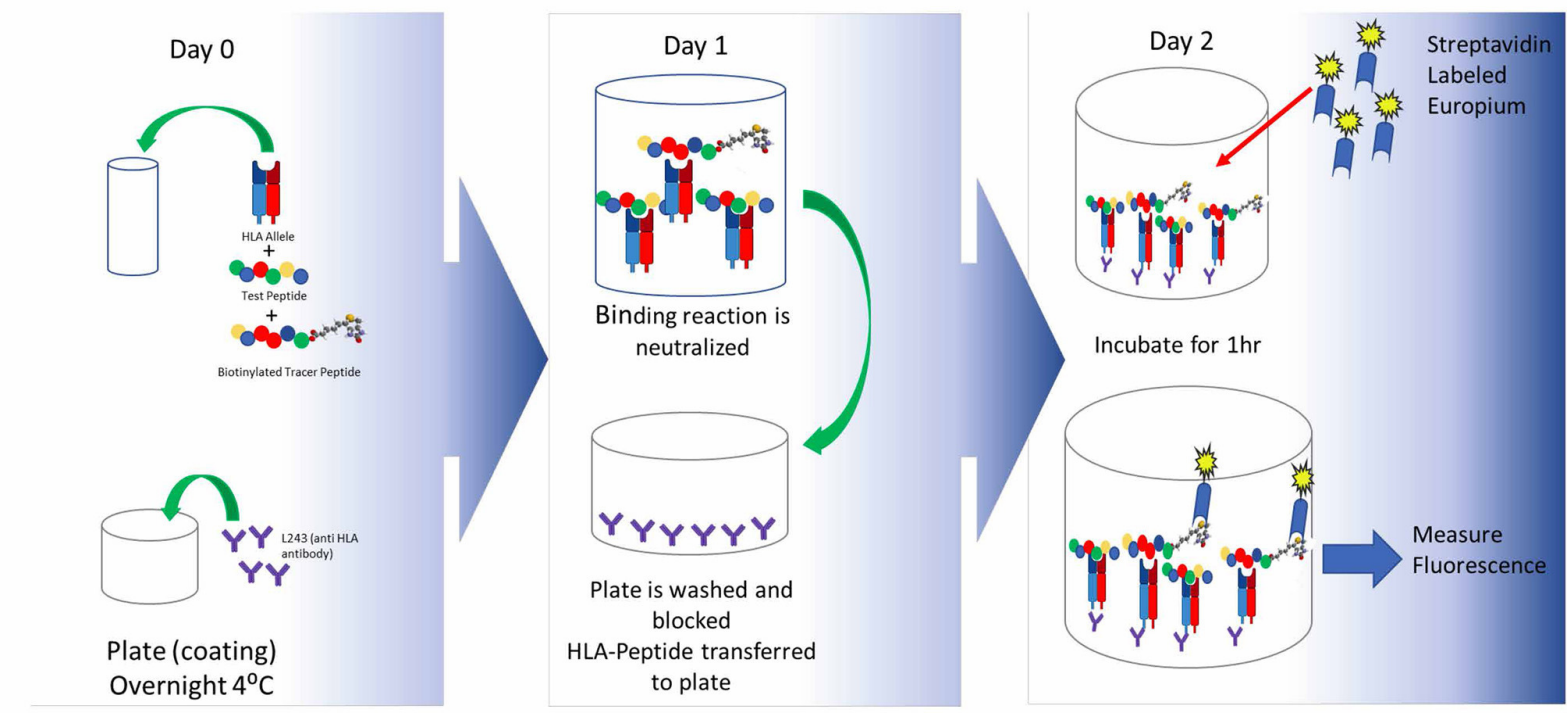
HLA binding assays and optimal peptide design. In brief, the peptide of interest is incubated with an allele-specific labeled tracer peptide and a soluble HLA supertype monomer are incubated to equilibrium. The following day the binding reaction is halted and the mixture is transferred to assay plates precoated with a pan anti-HLA-DR antibody and incubated overnight. Following this incubation, the plates are developed and peptide binding is indirectly measured by time resolved fluorescence spectroscopy. By using a fixed concentration of the labeled tracer peptide and a range of concentrations for the test peptide, one can generate a multi-point dose ranging curve that enables the calculation of an IC50 value which provides information not only about the ability of the peptide to bind HLA (yes/no) but also about the relative affinity of the peptide to a given HLA-DR supertype. Once can utilize the IC50 values to divide peptides into categories based on their affinity for a given HLA allele, such as high, moderate, low, and non-binding. As new technology becomes available and accessible, it will be useful to look at the kinetics of the binding reaction as well.

The first step in generating a T cell response is recognition of a peptide antigen presented on a HLA class II / MHC class II molecule to a T cell by an APC. Once a potential epitope is identified by *in silico* analysis, the prediction can be first validated through HLA binding assays, such as the assay described by Steere et al. (131), to assess the ability of a peptide to bind one or more HLA supertype alleles. Supertype alleles refers to families of HLA-DR alleles that share epitope binding motifs. By taking advantage of these supertype families, it is possible to perform binding assays on a relatively small number of alleles while covering >95% of the human population worldwide. A standard binding assay is described in Figure 3.

A key factor in generation of meaningful binding assay data is the design of the peptide sequence to be tested, and source of the test peptide. The core binding region of a class II peptide contains nine amino acids that sit within the peptide binding groove of an HLA molecule. This interaction is stabilized by flanking residues on either side of the core binding region and extend outside of the binding groove. When designing peptides for binding assays, it is important to properly center the binding motif within the peptide. Failing to do so can lead to the absence of binding despite the presence of an HLA binding motif. This is often seen in data generated by making use of overlapping peptides (83, 132).

**Figure 4:**
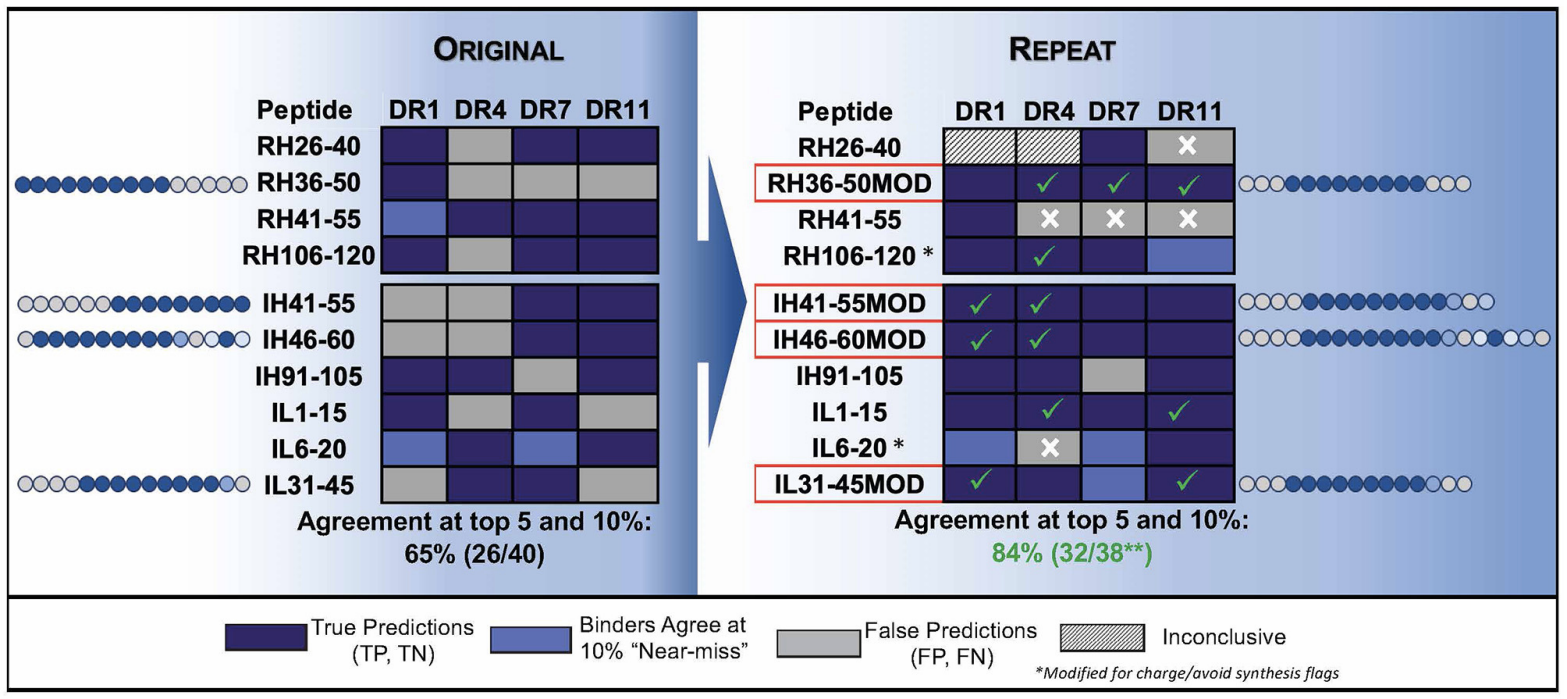
Optimizing test peptides for the HLA peptide binding assay. HLA binding data for Infliximab peptides, published by (83) are shown as described in the publication and compared to *in silico* predictions. The peptides were re-synthesized with centered HLA binding motifs and the assays were performed using a seven-point concentration curve in a competition assay. *In silico* predicted core residues are shown in dark blue and flanking residues predicted to stabilize binding but not to interact with the binding groove are shown in gray. Residue positions in source protein are indicated next to the results for HLA binding assays to four HLA DR alleles (Columns). **(Left)** Shows the results for HLA binding of original (15mer, overlapping by 5) peptides tested *in vitro* and published data, as compared to *in silico* predictions. The agreement between predicted and published is only 65%. **(Right)** Shows repeat data with optimized peptides (MOD) with centered HLA-binding motifs, and repeat assays using a more sensitive assay (competition assays, see Figure 2) as compared to *in silico* predictions. Centering the HLA binding motif and using a more sensitive assay improved the agreement between *in silico* and *in vitro* assays to 84%. Assay performed by BJR, peptides synthesized at Twenty-first Century Peptides, Waltham, MA).

The negative impact of improper centering of the T cell epitope in the peptide sequence (centered, with flanking residues on either side) is shown in Figure 4.

Peptide purity can also affect the outcome of a binding assay. Purity from some manufacturers can be as low as 60% due to the manufacturing process and the purity of the raw materials. Impurities within the peptides can lead to false positives and lead to faulty conclusions. Peptides for binding assays should be at a minimum 85% pure and should be ordered as net peptide. Spurious results can also be attributed to faulty synthesis. For example, non-binding peptides may have been synthesized on the same machine as earlier runs used to synthesize HLA binding peptides. This type of contamination can derail a drug development program, see for example, reference (133).

### *Ex vivo* Assays

#### PBMC Assays

Peripheral blood mononuclear cells (PBMCs) isolated from whole blood are the most prevalent source of responder cells for *in vitro* cell based assays for immunogenicity prediction (19, 29). The PBMCs used in experiments can be freshly isolated from healthy volunteer or diseased individuals or thawed from a cryopreserved bank of material potentially covering an appropriate representation of disease relevant or common well-documented HLA alleles. Due to the high throughput and ease of execution, PBMC assays using whole PBMCs, or CD8+ T cell depleted PBMCs remain the most commonly performed *in vitro* cell based assay for measuring the potential of immunogenicity (83, 119, 134–136).

In addition to typical biological products like protein, antibodies etc., product co-impurities including such as host cell proteins components, protein aggregates, synthesized peptide fragments, and others can also be evaluated in these assays. Multiple rounds of stimulation can be performed by replacing cell supernatants with fresh media spiked with the desired stimulant during extended culturing in order to expand populations of antigen specific T cells for further characterization (29, 119, 137). Schultz et al. recently reported success with a variation of the PBMC cell based assay that allows the enrichment of the number of CD4+ T cells prior to co-culture with irradiated syngeneic PBMCs in an effort to increase throughput and sensitivity (138).

The biological outcomes for T cell activation can be measured in these *in vitro* assays (both PBMC based and DC-T cell (see below) using a number of readouts. T-cell proliferation as assessed by thymidine incorporation and CFSE dye dilution are used frequently (7, 139, 140). Activation induced cytokine secretion may be measured using a focused (IL-2, IL-4, IFN-γ) or large multiplexed cytokine immunoassay panels and ELISPOT and are used as markers for T-cell activation and immunogenicity potential (136, 141, 142). Flow cytometry based detection of T cell responders allows a further characterization of the response in terms of intracellular cytokines, regulation of cell surface markers of activation, signal transduction events, and proliferation of specific T cell types (143, 144).

#### DC-T Cell Assays

*In vitro* co-cultures of monocyte derived dendritic cells (moDCs) and autologous CD4+ T cells are being increasingly used to evaluate immunogenicity potential of drug candidates and product CQAs. The DC-T cell or DC-PBMC methods pare the system down to the basic components of cell mediated immunity: CD4+ T cells interacting with an APC at relevant cell ratios, enhancing sensitivity as the total number of potential responder cells in the experimental system is much greater than the whole PBMC method. However, this method is time consuming and requires isolation and differentiation of monocytes into dendritic cells followed by an antigen loading/pulsing step which may be reagent, operator and material dependent.

Monocytes may be isolated from PBMC starting material using plastic adherence or isolation steps using magnetic bead separation methods. Differentiation and maturation of moDCs using cytokines or other factors is then performed (7, 57, 144–146), concurrently with the addition of the desired biotherapeutic, peptide fragments, or aggregates. The matured, pulsed moDCs are then typically combined in a co-culture with autologous, purified CD4+ T cells to allow for antigen presentation and T cell activation depending on immunogenicity potential. The responses are measured as is performed for PBMC assays as described above. An advanced variation of the moDC-T cell system is the Modular Immune *In vitro* Construct (MIMIC^®^) model which is capable of reproducibly generating both antigen-specific innate and adaptive immune responses against biologic such as proteins, peptides, mAbs as well as novel modalities including nucleic acids (147, 148) has also been described for these purposes.

#### Flow Cytometry Analysis of T Cell Phenotype

Flow cytometry has become a valuable tool in the assessment of immunogenicity that allows for the characterization of an immune response down to the single cell level (149). As the instruments become more sophisticated by adding more laser and filter combinations as well as advances in staining and detection methods, a wealth of information can be obtained from a sample of patient's blood.

T cell epitopes have the capacity to be either immunogenic or tolerogenic. While it may be difficult to measure the expansion of Tregs in cell culture, the presence of Treg epitopes can be confirmed by co-incubation with effector T cells in the presence of immunogenic peptides. In this “bystander assay,” activated Tregs inhibit the antigen-specific T effector response to the immunogenic peptides (150).

A standard bystander assay makes use of the immunologic memory toward antigens such as tetanus toxin, to which the majority of the population has had previous exposure through vaccination or natural exposure. PBMCs are cultured for 10 days in the presence of inactivated tetanus toxoid and the Tregitope at varying concentrations. Cells are stained for analysis by flow cytometry (Teff cells are defined as CD3+CD4+CD25+FoxP3low, Treg cells are defined as CD3+CD4+CD127lowCD25+FoxP3hi) and proliferation can then be measured by CFSE dilution. In the presence of Tregitope, we have observed a reduced proliferation of effector T cells to tetanus toxoid compared to the tetanus toxoid alone (151).

### Proteomics

#### MAPPS Assays

In the early 1990s an additional method called MAPPs was first described (152). This assay has proved valuable in identifying processed peptides presented on the surface of antigen presenting cells by relevant HLA. Additionally this approach attempts to understand the variability in antigen processing contributed by enzyme cleavages in healthy and diseased subjects and sequencing of the peptide associated with HLA can provide confirmation/validation to the sequences identified by algorithms.

Recent advancements in LC/MS sensitivity and proteomics analysis have enabled HLA bound mapping assays to be utilized pre-clinically to map potential antigenic sequence contained within a biological therapeutic. Studies have shown that not all potential HLA binding peptides are processed and presented by APC due to a combination of partial unfolding HLA binding and cathepsin trimming. Additionally, editing functions of HLA DM and HLA DO further enhance selectivity of the peptides selected for presentation (153).

**Figure 5:**
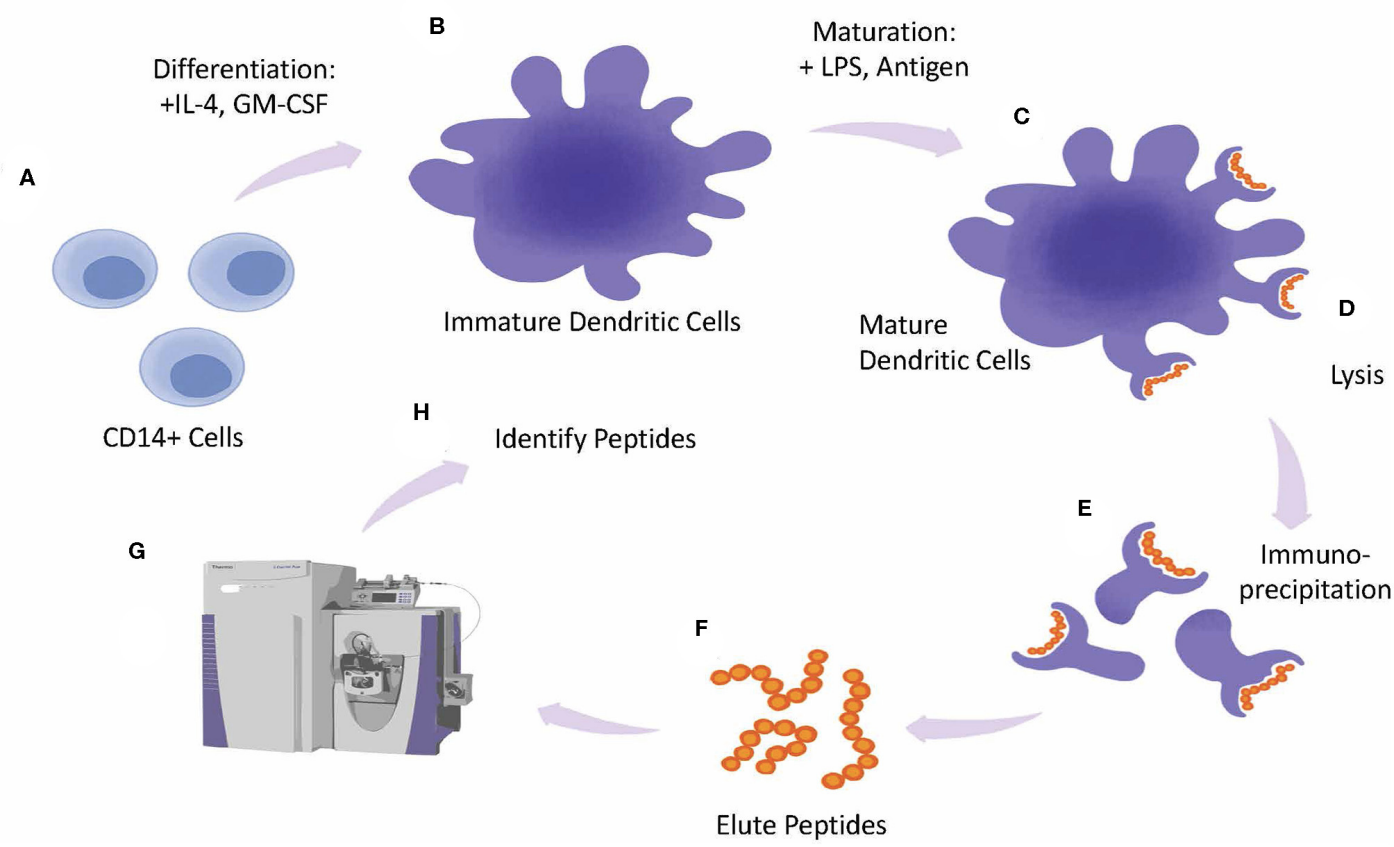
MAPPs assay design. Overview of MAPPS assay. Monocytes are isolated from whole PBMCs and differentiated into Dendritic Cells (DCs) in the presence of IL-4 and GMCSF **(A)**. Immature DCs are matured by incubating cells with LPS and antigen **(B)**. Mature DCs **(C)**, are lysed **(D)**. releasing peptide-loaded HLA molecules from the plasma membrane which are collected by immunoprecipitation **(E)**. Next peptides are eluted from the HLA molecules **(F)** and analyzed by Mass Spec **(G)**. Peptides are identified by screening them against a database of known antigens **(H)**.

In these assays (presented as a schematic in Figure 5) antigen presenting cells are generated *in vitro* and incubated with the therapeutic protein of interest for 24 h followed by a cytokine/mitogen induced maturation step to upregulate HLA expression. After cell lysis HLA receptor peptide complexes are isolated by immune precipitation followed by an acid elution step to dissociate the peptide from the HLA complex and sequenced by LC/MS. Subtraction of endogenous peptides and mapping of the peptides to the therapeutic can be done using proteomics protein database algorithms. These assays are likely to point toward antigenic peptides that can be targeted for deimmunizing protein engineering. Furthermore, whole blood from relevant diseased state can provide insights into altered presentation as well as tolerance for recombinant replacement therapeutics.

A case study showing the use of algorithms, innate and adaptive phase outputs as well as MAPPs was applied to anti-IL-21 receptor ATR-107 (144). *In silico* analysis of the primary sequence predicted two overlapping CD4 T cell epitopes in the heavy chain Complementary Determining Region (CDR) 2, and one single epitope in the light chain CDR2. The MAPPs confirmed the epitope in LC CDR2 as a dominant peptide presented by DCs. ATR-107 induced DC activation as attested by an increased expression of cell surface activation markers and cytokine production, and specific proliferation of autologous CD4 T cells in co-culture conditions. As illustrated in Figure 5, the validation of *in silico* predictions using MAPPS can be reassuring for developers.

However, elution of a peptide in a MAPPs assays does not confirm whether the peptide drives T-cell dependent immune response (13). T cell responses may differ depending on the phenotype of the T cells that are responding to the sequence. Using MAPPs without additional tools that explore the phenotype of T cells that respond to the eluted peptides, may over predict immunogenicity.

The importance of individual epitopes driving immunogenicity was also reinforced in a recent demonstration by Cassotta et al. who conducted a MAPPS analysis of natalizumab immunogenicity, a humanized antibody directed against alpha4 integrins (82). Taking advantage of a combination of *in silico* and in cellular *in vitro* assays, in particular a MAPPs assay performed with B cells isolated from patient peripheral blood, the authors established that two multiple sclerosis patients treated with Natalizumab who developed neutralizing ADA mounted a T cell response against a CD4 T cell epitope located in the V region of the light chain.

## Mitigation of Immunogenicity

### Mitigation by Deimmunization and Tolerization

#### Deimmunization

Ideally, mitigation of immunogenicity starts with the engineering of molecules designed to exhibit a low risk of provoking unwanted immune responses in patients. This can be achieved by combining the deimmunization and tolerization processes. In the case of monoclonal antibodies, deimmunization encompasses two non-mutually exclusive approaches: ultra-humanization, which consists of grafting murine CDRs into antibody frameworks of human origin, and removal of T cell epitopes sequences identified through the combination of epitope prediction logarithms and *in vitro* confirmatory assays. For examples of mitigation strategies involving the removal of T cell epitopes see (127, 154–156).

Grafting of murine CDRs into human V regions often leads to a decrease or loss of affinity, which can be restored by introduction of murine amino-acids in the human framework at positions critical for drug-target interactions. These so-called back-mutations have the potential to introduce additional T cell epitopes, hence the necessity to apply an iterative and timely deimmunization strategy to exhaust the possibilities of epitope removal as the sequence of the molecule is refined to reach the desired predicted efficacy. In this context, the Augmented Binary Substitution technology could prove an effective combinatory approach but needs further exploration (157).

#### Tolerization

Complementary to the removal of deleterious CD4 T cell epitopes is the introduction of T regulatory sequences, a process also known as tolerization (126). This is of particular interest in the case of replacement therapies, where removal of T cell epitopes might affect the function of the drug, or in the case of gene therapy to counterbalance the activation of the cytotoxic response induced by capsid antigenic determinants. Indeed, prophylactic administration of an AAV-derived capsid protein fused to Tregitopes was found to reduce viral capsid-specific CD8 T cell responses with a concomitant increase in Treg numbers (158). To date, the demonstration of the expected reduced immunogenicity of de-immunized and/or tolerized molecules relies on *in vitro* and *ex vivo* assays or re-clinical models (127, 154, 155, 159). De-immunized versions of high immunogenicity monoclonal antibodies have yet to reach the clinic, as biotherapeutics developers have focused instead on developing new, less immunogenic molecules that have a longer patent life and greater freedom to operate.

### Treatment-Induced Tolerance

Efforts to mitigate the risk of ADA development often focus on reducing therapeutic protein's intrinsic immunogenicity, with the exception of the well-established immune tolerance induction protocols for hemophilia A and B patients who develop inhibitors to recombinant clotting factors. ADA development to monoclonal antibody-drugs can also lead to loss of response and drug switching, even in the case of fully humanized molecules. In this context, various approaches to inducing immune tolerance to biotherapeutics have been envisaged and reviewed elsewhere (160). ADA responses to other lifesaving therapeutic proteins, such as enzyme replacement therapies, have compromised treatment efficacy and even caused death. In the case of gene therapy, development of ADA to the transgene and the viral vector remains major obstacles to treatment success: patients with pre-existing neutralizing antibody response to the viral capsid are not eligible for treatment, and patients who develop treatment-induced humoral immunity will not be eligible for re-dosing.

While removal of T helper epitopes that drive T helper immune responses may reduce T helper immune responses, in a process called deimmunization (127), identification and augmentation of Treg responses by preservation of Treg epitopes or introduction of Treg epitopes such as Tregitopes into the protein sequence is now referred to as “immune engineering” or “tolerization” (126). This *in silico* approach enables the introduction of regulatory T cell epitopes to reduce the potential for immunogenicity.

Alternatively, immune tolerance induction regimens can be undertaken using available drugs that target the major players of the immune cascade that leads to ADA development, by either inhibiting deleterious effector responses or activating tolerogenic pathways. The former can be realized by interfering with T and B activation mechanisms or by depleting immune cells with immunosuppressive agents such as cyclophosphamide or methotrexate, anti-CD3, anti-CD20 antibodies, proteasome inhibitors, or a combination of multiple depleting agents. Several such approaches are already in use, including concomitant methotrexate to diminish T cell-mediated immunogenicity (161, 162).

In Pompe disease and in the context of tolerance induction for inhibitors to FVIII therapy, current regimens combine multiple agents such as Rituximab (to eliminate antibody-secreting B cells) and IVIG (to bind and remove antibodies or to induce tolerance). Methotrexate is added in Pompe disease, and this regimen has been successful in establishing tolerance to alglucosidase alpha in high risk Pompe disease infants (163).

Other methods under consideration include concomitant administration regimen of rapamycin in a nanoparticular form (164–167) or co-administration with Tregitopes (48, 168). Infusion of *in vitro* expanded T and B regs engineered to express antigen-specific receptors was also shown to control development of inhibitors in a pre-clinical model of hemophilia A (169).

Most immune tolerance induction approaches are still at an early stage of development, and the long term impact of these interventions remains unknown. However, the demonstrated value of the tolerizing regime that have reached the clinic is an incentive to pursue the evaluation of immune tolerance induction as a mean to mitigate unwanted immunogenicity of biotherapeutics.

## Future Directions

### ADA Assay Standardization

Comparing immunogenicity of therapeutic proteins across clinical studies has proven difficult due to the lack of ADA assay standardization and harmonization. For a given therapeutic protein, variability in critical assay parameters such as sensitivity and drug tolerance can lead to dissimilar estimation of clinical incidence across laboratories. In this context, the IMI-funded ABIRISK consortium (Anti-Biopharmaceutical immunization: prediction and analysis of clinical relevance to minimize the risk) generated monoclonal antibodies to serve as standards in ADA assays. Such universal standards could be used to benchmark assay sensitivity and drug tolerance, monitor routine assay performance, and validate antigenicity equivalence of comparator products in biosimilars ADA assays. Additionally, the immunogenicity assessments with such standards can help inform the clinician on dosing strategies if loss of efficacy is observed (170). Monoclonal neutralizing antibodies of various isotypes and affinity specific for rituximab, natalizumab, infliximab, adalimumab, or Interferon beta were generated from B-cells isolated from patients immunized with the respective therapeutic proteins, as previously described (171). Production scale-up and further characterization using ABIRISK validated ADA assays are on-going. Ultimately, all antibodies will be openly available at the National Institute of Biological Standards and Controls (NIBSC).

### New Modalities and Immunogenicity Risk Assessment

**Figure 6:**
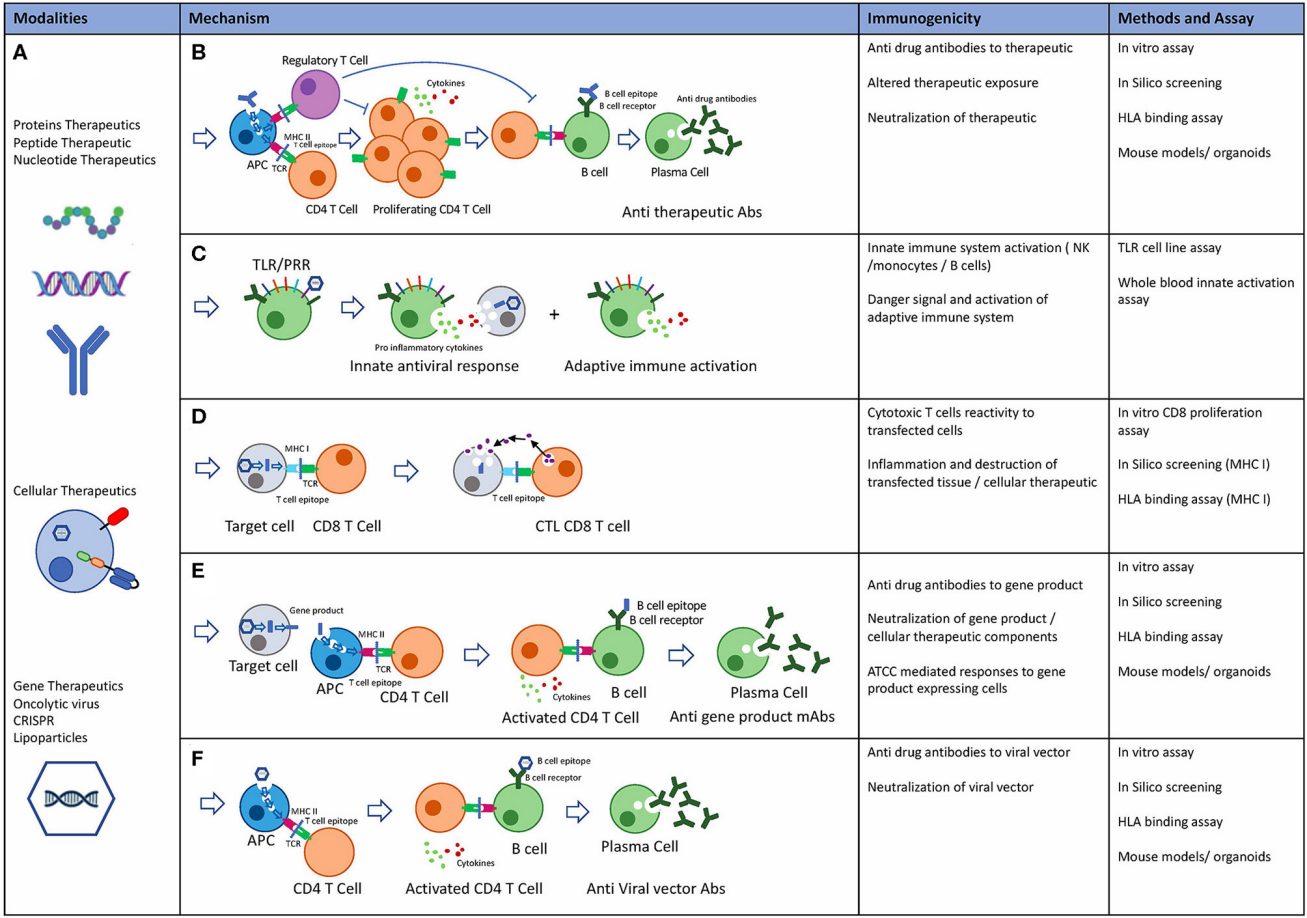
Novel Biological modalities, potential immunogenicity mechanisms and pre-clinical risk assessment tools. **(A)** Protein therapeutics: including monoclonal antibodies, peptides, endogenous proteins, RNA, and DNA based therapeutics. Cellular therapeutics including CAR-T TCR-T and other engineered cells including delivery systems. Gene therapeutics including virally delivered genes, oncolytic virus therapeutics, CRISPR gene therapy, and delivery systems. **(B)** Potential mechanisms of immunogenicity: Adaptive immune response is an HLA class II mediated immune response to exogenous antigen resulting in Anti therapeutic antibody. Immune suppressive effects through T regulatory cells mediated tolerance to biological therapeutics. **(C)** Innate immune system: Activation of the innate immune system may occur through TLR and PPR receptors on immune cells in response to exogenous proteins and particles. Inflammatory cytokine release and adaptive immune system activation through danger signals. **(D)** Cytotoxic T cell (CTL) mediated adaptive immune response drives immunity to intracellular proteins or expressed gene products. **(E)** Adaptive immune response may occur to gene products that are secreted, expressed or taken up by APC after cell death resulting in anti-drug antibodies to gene products. **(F)** Preexisting anti capsid mAbs may be present due to previous exposure to viruses. Alternatively, adaptive immune response can also be targeted at viral capsids.

As discussed in section New Modalities, new modalities such as cellular and gene therapies have shown immunogenicity in the clinic. The mechanisms by which these modalities can elicit immune response are complex due to the high level of engineering, intracellular expression, introduction of engineered gene products, as well as complex delivery systems. Modified Immunogenicity risk assessment tools and assays developed primarily for protein therapeutics can be used to minimize immunogenicity risk of these novel therapeutics (Figure 6).

### Specific Cell Lines/Soluble TCRs

Novel *in vitro* assays that rely on the ability of antigen presenting cells displaying the processed peptides in the context of HLA class I/II to interact with T cell repertoires are proving to be useful for further defining the antigen specificity and immune response propagation (172, 173). Additionally, use of engineered B-cell lines expressing class I and class II HLA can support a high-throughput prediction of intracellular processing and presentation of potential antigenic epitopes. One example would be to use a competitive approach where soluble T cell receptors recognizing an HLA-reference peptide complex are used to detect presentation of potential immunogenic epitopes by mono-allelic antigen presenting cell lines (Merck, unpublished data).

### Modeling

As described above, a suite of *in silico* and *in vitro* tools can be deployed early in development to guide protein engineering and design drug candidates with predicted low immunogenicity. However, the tools will assess product-related risks, in particular sequence-based risk, but won't inform other factors pertaining to immunogenicity such as patient- and treatment-related factors. The overall immunogenicity risk relies on the weighting and integration of the different risks, some of which are empirical, some theoretical. Immunogenicity Quantitative Systems Pharmacology (QSP) simulators could simplify and homogenize this integration (174). They incorporate biotherapeutics, physiologically-based pharmacokinetic, and mechanistic models of immune responses to simulate large scale clinical trials and predict immunogenicity incidence. The impact of critical variables such as HLA genotype, combination therapies, dosing regimens and route of administration on ADA incidence, as well as ADA impact on drug Pharmacokinetics (PK) can be modeled. QSP simulators are still in development, requiring a greater set of empiric input data and refinement of parameters related to the immune system such as kinetics of antibody development (174). Once validated, QSP simulators could give rise to personalized management and mitigation of immunogenicity.

## Discussion

### Immunogenicity-Focused Organizations

Faced with the challenge of accurately performing an immunogenicity risk assessment as well as measuring and determining the clinical relevance of ADA, pharmaceutical companies, biotech and contract research organizations joined forces to progress the field by addressing the gaps. Scientific non-profit associations were created, such as the European Immunogenicity Platform (EIP, https://www.e-i-p.eu/). The purpose of the EIP is to stimulate exchanges between immunogenicity experts, encourage, and lead interactions with regulatory agencies, share knowledge and state-of-the art in immunogenicity field with the broader scientific community and training courses on practical and regulatory aspects of immunogenicity.

The ABIRISK consortium mentioned above represents another collaborative approach to contributing to the advancement of immunogenicity sciences. Clinical and basic research academic centers worked with industrial partners on a 6-year research project, addressing some of the main questions and practical hurdles related to unwanted immunogenicity, such as the value of existing predictive tools, ADA assays, harmonization and standardization, clinical relevance of detected ADA, identification of patients' risk factors, and predictive markers (175–178).

A spin-off initiative emerged from this extensive collaboration across laboratories in Europe, the United-States and Israel. BIOPIA (https://ki.se/en/cns/biopia) is a non-profit effort of European laboratories with expertise in biopharmaceutical pharmacokinetics and immunogenicity in many diseases, which aim to raise awareness about immunogenicity and advocate integration of drug levels and ADA testing as a means to improve patient's management. The website provides information about ADA and drug level testing with the goal of helping clinicians with the implementation of routine, clinical testing for immunogenicity and drug levels. Similar efforts are underway in the United States, under the umbrella of the Therapeutic Protein Immunogenicity Community as part of the AAPS (American Association of Pharmaceutical Scientists). The Immunogenicity risk assessment and mitigation (IRAM) working group has initiated a survey to characterize performance and harmonize methods for risk assessments including algorithms and *in vitro* assays through member surveys. The future focus is on adapting the current tools and developing innovative assays to answer questions around novel modalities and next generation therapies.

### Regulatory Perspective on Immunogenicity

The recent FDA guideline proposes a risk-based approach to assess immune response to a therapeutic protein and its impact on safety and efficacy on a case-by-case basis (33). There is also a recommendation that the risk-based strategy be developed early in development, preferably after humanization and in parallel with other developability efforts. The early assessment would enable a more robust understanding of the liabilities due to structure and sequence. A continuous evaluation of the risk through the different stages of drug development can guide the bioanalytical strategy for clinic as described below. This would include risks due to changes in process development, manufacturing, formulation, and device.

### Integration of Risk Assessment Into the Preclinical Pipeline

Briefly, the immunogenicity risk assessment should take into account potential therapeutic benefits and weigh those against the potential impact of immunogenicity taken into account patient population and indication as well as previous experience with therapeutic target.

Early assessment of biologic candidates allows ranking based on least probability of identified risk. There is also room for deimmunization/sequence optimization which could involve removing a few amino acids to remove the epitope or inserting regulatory sequences to drive a suppressive T cell response. Furthermore, the risk-based strategy should include any liabilities due to post-translational modifications that are a consequence of process related changes associated with expression, purification, etc. as well as formulation/excipient induced aggregation or degradation.

**Figure 7:**
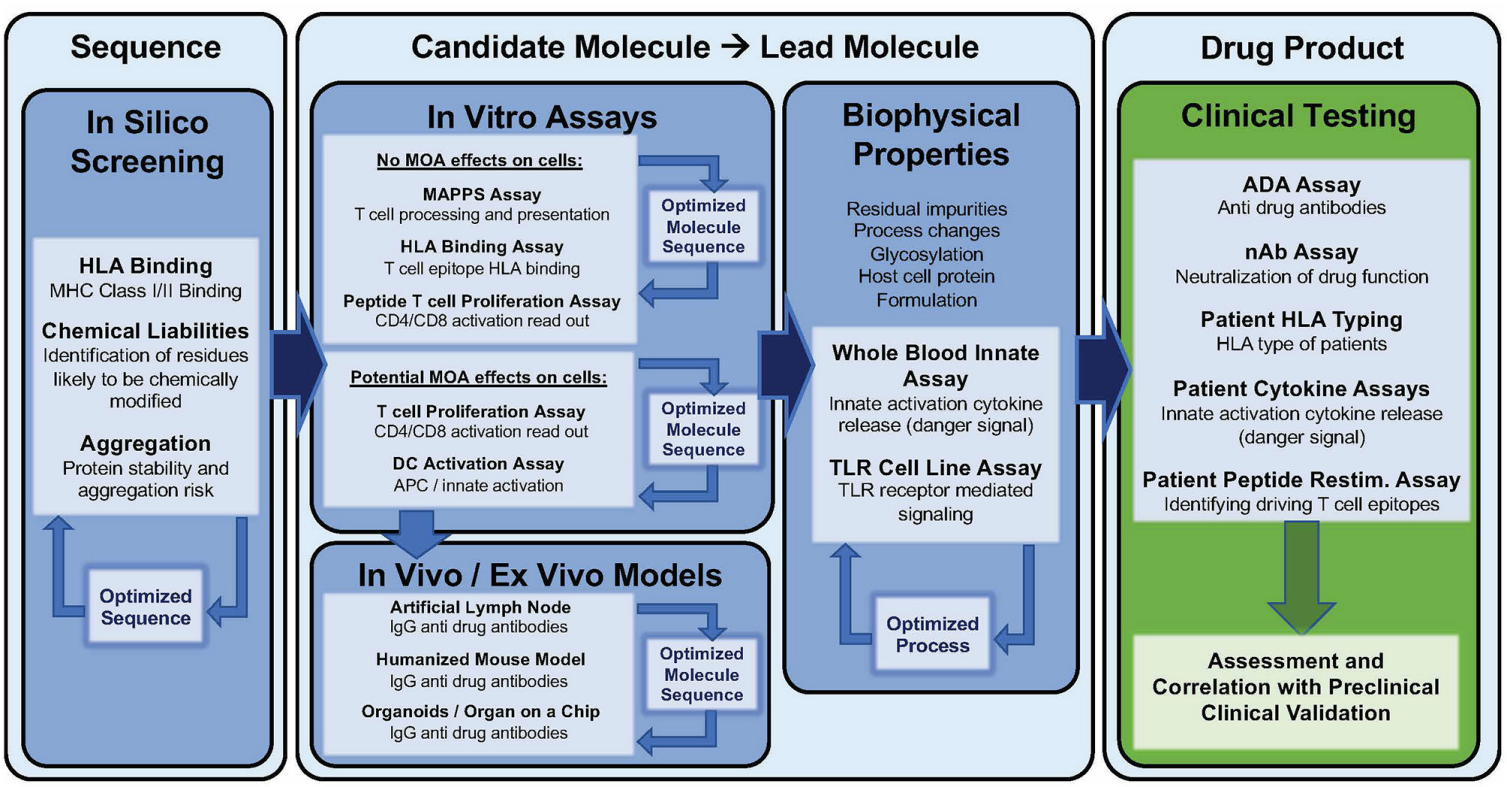
Immunogenicity risk assessment tools in biological drug development. Tools and assays that can be utilized at different stages of lead candidate selection to minimize immunogenicity risk. For example, ***In silico* screening:** computer-based algorithms can evaluate amino acid sequence for potential HLA class I and II binding, residues that are likely to be chemically modified and assess the of the protein structure to aggregate. ***In vitro* assays** can be utilized to assess the potential of biological therapeutics to elicit activate T cells in diverse donor sets. These assays can be performed with whole protein to potentially include target engagement or using overlapping peptides to exclude. **MAPPS and HLA binding assays** can be used to identify antigens within the molecule. ***Ex vivo* and** ***in vivo* models** encompassing additional compartments of the immune system can be used when specialized questions arise during development. **Innate immune activation assay** to evaluate the impact of non-sequence biophysical parameters can be used to optimized process development and formulation or process changes. **Clinical immunogenicity data** and patient characterization is a critical component to validate, evaluate and improve preclinical tools and assay.

The knowledge of early pharmacology of the therapeutic protein including on and off -target engagement and consequent activation of the immune pathways should also be a consideration during development of the risk-based strategy. This is especially relevant for therapeutic proteins (TPs) targeting immune modulatory pathways. The pre-clinical toxicology studies could provide an insight into the safety associated concerns related to on and off target liabilities, especially when the pre-clinical and clinical targets have homology. The risk-based strategy can also benefit, where there is previous clinical experience such as proteins with similar targets that are already in the commercial phase. Additionally, if there is enough clinical experience around the TP in one disease indication, the outcome of the studies related to any safety and efficacy can also be summarized for the investigational new drug application (IND) being developed for the new IND. Figure 7 provides an overview of the sequence to product stage of development and tools and their outputs to address key attribute relate questions.

## Five Year View

As a result of advances in immunogenicity risk assessment methods as well as derisking efforts pertaining to both the product (primary sequence and formulation) as well as improved understanding of the patient factors that may contribute to development of ADA, most biotherapeutics developers are integrating the assessments into pharmacokinetics/pharmacodynamics, safety and clinical efficacy outcomes to better understand the risk of a new product or biosimilar. Ongoing consideration should be given to use of emerging technologies (novel *in silico, in vitro*, and *in vivo* assays) for use during development (designing of new sequences, lead selection, de-risking of identified liabilities, or comparison of biosimilar prioritization). These methodologies also provide an estimation of risk, including prior knowledge of individual risk (HLA type) disposition for clinical immunogenicity. *In vivo* studies in animal models are not currently recommended for immunogenicity due to differences between animal model and human HLA. Instead, *in vitro* assays are preferred for evaluating risk of cell-mediated immune responses. MHC-related immune response variation can be expected when transitioning from one model species to another, or to human. T cell epitopes bound by MHC in mice, non-human primates, and other model species are frequently different than those bound by humans. Testing for immunogenicity *in vitro*, with human PBMC samples that are selected to provide broad coverage of human MHC, is how most pre-clinical studies with biologics circumvent this concern.

Within 5 years, it is expected that much of the risk-assessment will be performed first *in silico* before moving to (limited) *in vitro* and *in vivo* models. This is due to the fact that most drug companies performing comprehensive pre-clinical development generate literally thousands of potential candidates for a single target. *In silico* analysis gives a good first pass approach to immunogenicity, enabling detailed inspection of certain molecular features using *vitro* methods where required. The accuracy of computational tools will increase with increasing results available to public review.

Machine to machine interfaces, enabling the integrated and high throughput screening of multiple candidates for the same target, will simultaneously improve the pre-clinical selection of candidates for clinical development. Drug developers will need to become familiar with available tools as the sheer volume of candidates that are expected to be screened will be impossible to manage without automated *in silico* analysis pipelines. It is also likely that the breadth of *in silico* analysis (and *in vitro* validation) will begin to encompass HLA class I immunogenicity assessment and *in vitro* assays. This is due to the introduction of novel modalities and viral vectors, which interface with the class I pathway.

The field of immunogenicity risk assessment has matured and will continue to evolve as new modalities are introduced into the clinic.

## Author Contributions

VJ, FT, JG, SM-K, BR, ST, and AD conceptualized this review, composed, and edited the manuscript document. Figures were created by JG, BR, and FT. All authors contributed to the article and approved the submitted version.

## Conflict of Interest

AD is a senior officer/majority shareholder and BR and FT are employees at EpiVax, Inc., a privately owned immunotherapeutics company located in Providence RI. These authors acknowledge that there is a potential conflict of interest related to their relationship with EpiVax and attest that the work contained in this report is free of any bias that might be associated with the commercial goals of the company. JG is an employee of Bristol Myers Squibb and is a shareholder of Bristol-Myers Squibb. This author acknowledges that there is a potential conflict of interest related to their relationship with Bristol Myers Squibb and attests that the work contained in this report is free of any bias that might be associated with the commercial goals of the company. VJ is currently an employee of Merck and Co. and owns Merck and Co. stock. This author acknowledges that there is a potential conflict of interest related to their relationship with Merck and attests that the work contained in this report is free of any bias that might be associated with the commercial goals of the company. ST is currently an employee of Pfizer. This author acknowledges that there is a potential conflict of interest related to their relationship with Pfizer and attests that the work contained in this report is free of any bias that might be associated with the commercial goals of the company. SM-K is employed by and has a stockholder interest in Sanofi. This author acknowledges that there is a potential conflict of interest related to their relationship with Sanofi and attests that the work contained in this report is free of any bias that might be associated with the commercial goals of the company.

## Abbreviations

AAV: Adeno-Associated Virus
ADA: Anti-Drug Antibodies
ADC: Antibody Drug Conjugates
APC: Antigen Presenting Cell
API: Active Pharmaceutical Ingredient
CAR: Chimeric Antigen Receptor
CDR: Complementary Determining Region
CPI: Check Point Inhibitors
CTL: Cytotoxic (Usually CD8+) T cells
DAMP: Danger Associated Molecular Patterns
DC: Dendritic Cell
EPO: Erythropoietin
HCP: Host Cell Protein
HLA: Human Leukocyte Antigens
IND: Investigational New Drug application
IgG: Immunoglobulin G
IIRMI: Innate Immune Response Modifying Impurities
ISPRI: Interactive Screening and Protein Reengineering Interface
GAA: Acid Alpha Glucosidase
MAPPS: MHC-Associated Peptide Proteomics
MHC: Major Histocompatibility Complex
moDC: Monocyte-derived Dendritic Cells
NLR: NOD-Like Receptors
PRCA: Pure Red Cell Aplasia
PRR: Pattern Recognition Receptors
RA: Rheumatoid Arthritis
TCR: T Cell Receptor
Td: T cell dependent antibody response
Thelper: CD4+ helper T cells
Ti: T cell independent antibody response
TLR: Toll Like Receptors
Treg: Regulatory T cells
Tregitope: Regulatory T cell epitope in IgG.
